# The times are they a-changin’? Tracking sovereign wealth funds’ sustainable investing

**DOI:** 10.1057/s42214-023-00161-4

**Published:** 2023-05-16

**Authors:** Bernardo Bortolotti, Giacomo Loss, Robert W. van Zwieten

**Affiliations:** 1 SIL, Baffi Carefin Center, Bocconi University, Milan, Italy; 2 TIL, NYUAD, NYU Abu Dhabi, Experimental Research Building (C1), Saadiyat Campus, Abu Dhabi, United Arab Emirates; 3 Asian Institute of Management, 123 Paseo de Roxas, Legazpi Village, Makati, 1229 Metro Manila, Philippines

**Keywords:** sovereign wealth funds, Sustainable Development Goals, ESG

## Abstract

This paper analyzes the orientation towards sustainable investment by global sovereign wealth funds (henceforth SWFs) over the last two decades. Our data analysis reveals that over the last 5 years, there has been a noticeable uptick in investing along the Sustainable Development Goals (henceforth SDGs) by SWFs. From 2018 onwards, momentum has been building in climate and energy, especially in deal value. We also see agriculture come to the fore in 2020, and to a lesser degree, investments in education, as SWFs appear to extend their investment remits slowly but surely into other long-term investment themes. The paper also provides preliminary descriptive evidence about the drivers of sovereign sustainable investment, showing that the presence of explicit environmental, social, and governance (henceforth ESG) policies in place favor capital deployment aligned with SDGs. Finally, the paper studies the ESG performance of a sub-sample of listed firms that SWFs have invested in, finding a significant deterioration in the governance pillar. This result is broadly consistent with previous research on the agency costs of sovereign ownership. The paper concludes by making policy recommendations regarding fiduciary duty, investee corporate governance, and climate investments, which would contribute to modernizing the role of SWFs in the global economy.

## Résumé

Cet article analyse l'orientation des fonds souverains mondiaux vers l'investissement durable au cours des deux dernières décennies. Notre analyse des données révèle qu'au cours des cinq dernières années, les fonds souverains ont sensiblement augmenté leurs investissements dans le cadre des objectifs de développement durable (ODD). À partir de 2018, l'élan se renforce dans les domaines du climat et de l'énergie, en particulier en ce qui concerne la valeur des transactions. Nous voyons également l'agriculture occuper le devant de la scène en 2020 et, dans une moindre mesure, les investissements dans l'éducation. Les fonds souverains semblent étendre lentement mais sûrement leurs attributions d'investissement à d'autres thèmes d'investissement à long terme. Le document fournit également des données descriptives préliminaires sur les moteurs de l'investissement souverain durable, montrant que la présence de politiques environnementales, sociales et de gouvernance (ESG) explicites en place favorise le déploiement de capitaux alignés sur les ODD. Enfin, le document étudie la performance ESG d'un sous-échantillon d'entreprises cotées en bourse dans lesquelles les fonds souverains ont investi, et constate une détérioration significative du pilier de la gouvernance. Ce résultat est globalement cohérent avec les recherches antérieures sur les coûts d'agence de l'actionnariat souverain. L'étude conclut en formulant des recommandations politiques concernant l'obligation fiduciaire, la gouvernance d'entreprise des bénéficiaires des investissements et les investissements climatiques, qui contribueraient à moderniser le rôle des fonds souverains dans l'économie mondiale.

## Resumen

Este artículo analiza la orientación hacia la inversión sostenible de los fondos globales de riqueza soberana (SWFs en adelante) durante las dos últimas décadas. Nuestro análisis de datos revela que en los últimos cinco años se ha dado un remonte en la inversión de los SWFs junto a los Objetivos de Desarrollo Sostenible (SDGs, en adelante). A partir de 2018, se ha dado un ímpetu en clima y energía, especialmente en el valor del trato. También vemos cómo la agricultura toma protagonismo en el 2020, y en menor grado, inversiones en educación, ya que los SFWs aparentemente extienden el cometido de sus inversiones, de manera lenta pero segura hacia otros temas de inversiones a largo plazo. El artículo ofrece, además evidencia preliminar descriptiva de los propulsores de las inversiones soberanas sostenibles, demostrando que la presencia de políticas vigentes explicitas de Medioambiente, Social y Gobernanza (ESG en adelante) favorecen el despliegue de capital en línea con los SDGs. Finalmente, el artículo estudia el desempeño de ESG de una submuestra de compañías cotizadas, en las cuales SWFs han invertido y que identifica un deterioro significativo del pilar de la gobernanza. Estos resultados son ampliamente consistentes con investigaciones previas sobre los costos de agencia de la propiedad de soberanía. El artículo concluye haciendo recomendaciones de política que tienen que ver con la obligación fidusiaria, gobernanza corporativa participada e inversiones en clima, las cuales podrían contribuir a modernizar el papel de los SWFs en la economía global.

## Resumo

Este artigo analisa a orientação para o investimento sustentável por parte dos fundos de riqueza soberanos globais (SWFs para sua sigla em inglês) nas últimas duas décadas. Nossa análise de dados revela que, nos últimos cinco anos, houve um aumento notável no investimento dos SWFs alinhado aos Objetivos de Desenvolvimento Sustentável (ODS). A partir de 2018, o ímpeto está crescendo em clima e energia, especialmente em valor de negócios. Também vemos a agricultura ganhar destaque em 2020 e, em menor grau, os investimentos em educação, já que os SWFs parecem estender suas atribuições de investimento lenta mas seguramente para outros temas de investimento de longo prazo. O artigo também fornece evidências descritivas preliminares sobre os antecedents do investimento soberano sustentável, mostrando que a presença de políticas ambientais, sociais e de governança explícitas (doravante ESG) favorece a aplicação de capital alinhada com os ODS. Por fim, o artigo estuda o desempenho ESG de uma subamostra de empresas listadas nas quais os SWFs investiram, encontrando uma deterioração significativa no pilar de governança. Esse resultado é amplamente consistente com pesquisas anteriores sobre os custos de agência da propriedade soberana. O documento conclui fazendo recomendações de políticas em relação ao dever fiduciário, governança corporativa da empresa investida e investimentos climáticos, o que contribuiria para modernizar o papel dos SWFs na economia global.

## Abstract (zh)

本文分析了过去二十年来全球主权财富基金(SWFs)对可持续投资的取向。我们的数据分析显示，在过去五年中，主权财富基金在实现可持续发展目标（SDGs）方面的投资显著增加。从2018年开始，气候和能源领域的势头正在增强，尤其是在交易价值方面。我们还看到，农业在2020年开始引起注意，教育投资在较小程度上也引起注意，因为主权财富基金似乎缓慢但肯定地将其投资范围扩展到其他长期投资主题。本文还提供了关于主权可持续投资驱动因素的初步描述性证据，表明了明确合适的环境、社会和治理（ESG）政策有利于与可持续发展目标相一致的资金部署。最后，本文研究了主权财富基金投资的上市公司的子样本的ESG绩效，发现了治理方面显著的恶化。这一结果与之前关于主权归属的代理成本的研究大致一致。本文最后提出了关于信托责任、被投资公司治理和气候投资的政策制定建议，这将有助于实现主权财富基金在全球经济中作用的现代化。

## Abstract (de)

Dieser Beitrag analysiert die Ausrichtung der globalen Staatsfonds (nachfolgend SWFs) auf nachhaltige Investitionen in den letzten zwei Jahrzehnten. Unsere Datenanalyse zeigt, dass in den letzten fünf Jahren ein deutlicher Anstieg der Investitionen von Staatsfonds zugunsten der Ziele für nachhaltige Entwicklung (nachfolgend SDGs) zu verzeichnen war. Ab 2018 nimmt die Bedeutung von Klima- und Energieinvestitionen zu, insbesondere im Hinblick auf den Transaktionswert. Wir sehen auch, dass die Landwirtschaft 2020 in den Vordergrund rückt, und in geringerem Maße auch Investitionen in Bildung, da SWFs ihre Investitionsvorhaben langsam aber sicher auf andere langfristige Investitionsthemen auszuweiten scheinen. Der Beitrag liefert auch erste deskriptive Erkenntnisse über Faktoren, die nachhaltige Investitionen begünstigen, und zeigt, dass das Vorliegen von expliziten Richtlinien für Umwelt, Soziales und Unternehmensführung (nachfolgend ESG) einen an den SDGs ausgerichteten Kapitaleinsatz begünstigt. Schließlich untersucht der Beitrag die ESG-Performance einer Teilstichprobe börsennotierter Unternehmen, in die SWFs investiert haben, und stellt eine deutliche Verschlechterung der Unternehmensführungs-Säule fest. Dieses Ergebnis deckt sich weitgehend mit früheren Forschungsergebnissen zu den Agency-Kosten von Staatseigentum. Der Beitrag gibt abschließend politische Empfehlungen in Bezug auf treuhänderische Pflichten, die Unternehmensführung von Investitionsempfängern und Klimainvestitionen, die zur Modernisierung der Rolle von SWFs in der Weltwirtschaft beitragen würden.

## INTRODUCTION

With portfolios worth more than $10 trillion, SWFs are a prominent and fast-growing class of institutional investor. Given the sheer size of their assets, sovereign wealth funds can move the needle in achieving UN Sustainable Development Goals (SDGs) and bridge the huge financing gap developing countries face.^1^ Furthermore, state sponsorship legitimizes them to address market failures in their investment strategies, accounting for externalities, and investing in public goods. The intergenerational nature of SWF’s business places them in a better position to assess the materiality of long-term risks, such as climate change, to their portfolios. At the same time, as universal owners with large stakes in companies across a huge range of sectors and markets, SWFs are uniquely placed to drive the transition across the investment cycle through active and responsible ownership.

Yet, SWFs are often referred to as “sustainability laggards”, and their participation in the responsible investing movement has been claimed to be lackluster relative to other institutional investors and private-sector counterparts. According to a recent survey, only 13% of SWF interviewed had published a sustainability report in 2019, while the share of pension funds doing so is 31% (UNCTAD, 2020). Another survey on global asset owners shows that SWFs record the highest percentage (52%) of respondents declaring that they do not include ESG in their investment approach (Hentov, 2019). Turning to specific global challenges, most SWFs agree that climate change will affect economic growth and financial return, but 60% of respondents declare they are not taking climate-related risks and opportunities into consideration in the investment process in any systematic way (IFSWF, 2020).

In this large and heterogeneous group, Norway’s Government Pension Fund Global (GPFG), the largest savings SWF around the world, with a portfolio of USD 1.3 trillion entirely invested abroad, has been a frontrunner in responsible investing. The fund has pioneered negative screening, a process that excludes stocks in sectors conflicting with the fund’s strict ethical standards and divests companies caught, for example, in human rights violations or causing severe environmental damage. Within the same echelon of savings funds, the New Zealand Superannuation Fund (2020) is recognized as a global leader among institutional investors for having developed one of the most sophisticated strategies for combating climate change.

But the above-mentioned cases are notable exceptions. SWFs are generally portrayed as isolated institutions, shielded from the external pressure to change investment policies and deliver on the SDGs. Asked whether their boards and beneficiaries ask about such issues, only 38% SWFs say that they do, compared to around two-thirds of central banks, foundations, and endowments, and just over half of pension funds who take environmental sustainability into consideration (Hentov, 2019).

Over and above this anecdotal evidence, this paper aims to set the record straight about sustainable investment by global SWFs, by providing updated and comprehensive data about their deal-making in this space. We track two decades of SDG investments by SWFs, adopting UNEP’s broad definition of “sustainability”, which contains both environmental and economic inclusivity dimensions, and labeling SWF deals as sustainable (SDG) when they are executed in the sectors aligned with the IRIS+ taxonomy, a standard reference in the field.^2^

The data show that indeed SWF did not engage deeply in making sustainable investments, even if a trend is picking up since 2017. The sectoral distribution shows an impressive concentration in healthcare, and an even allocation among main target developed economies, while within emerging countries Southern Asia sticks out as the primary target due to the impressive activity of Singaporean funds. Sadly, Africa, the continent starving for this type of capital, is still under the SWFs’ radar.

While the paper remains mainly descriptive, we also try to study empirically the determinants of SWF sustainable investing in the framework of competing theories of the drivers of ESG considerations in investment decisions. While the small size of our sample limits the explanatory power of our tests, we find that political factors or SWF type in terms of developmental orientation do not seem to matter, while stronger ESG policies in place seem conducive to more sustainable investments by SWFs.

We also ask the question as to whether SWFs’ investment leads to an improvement in the ESG performance of the target firms. By analyzing a subsample of listed firms, we discover that, on the contrary, the ESG scores of investee firms tend to progressively deteriorate in the post-acquisition period. By investigating the individual pillars of ESG performance, we find that most of the decline is concentrated in the governance dimension, while environmental and social performance is barely affected. This descriptive analysis does not allow drawing any causal relation between SWF investment and the ESG outcomes of target firms. However, these findings are broadly consistent with the view that SWFs could negatively affect corporate governance by adopting a passive stance creating agency costs (Bortolotti, Fotak, & Megginson, 2015; Chen, Wei, & Dai, 2022a).

This paper is closely related to the growing literature on the environmental, social and governance (ESG) investing by SWFs. Liang and Renneboog (2020) study whether ESG considerations affect their investment decisions in a large sample of global publicly listed corporations. They find that the level of past ESG performance and as well as recent ESG score improvement are strong predictors of SWFs’ decision to take ownership stakes in listed companies. More specifically, results are driven by the SWF funds that do have ESG policy in place and are most transparent, and by SWF originating from developed countries and countries with civil law origins. In a similar vein, Dai, Song, You and Zhang (2022) show that controlling for firm characteristics, SWFs are more likely to invest in a US-listed company with higher Kinder, Lydenburg, and Domini (KLD) scores, a widely used ESG measure. Chen et al., (2022a; Chen, El Ghoul, Guedhami, & Liu, 2022b) analyze Chinese public equity markets and show that ESG factors positively and significantly help attract SWF investments in listed firms. Vasudeva, Nachum and Say (2018) analyze signaling effect of socially responsible investments of Norway’s GPFG, the largest SWF around the world. The authors show that GPFG’s screening ability of one country’s institutional quality unlocks international investment by conational firms. Indeed, internationalizing companies from Norway and Sweden are more likely to take larger equity commitments in firms headquartered in host countries where Norway’s SWF holds larger investment.

We contribute to this literature by providing new evidence on the determinants of SWF total investments, considering the very large realm of unlisted companies as well. In fact, private markets account for the overwhelming majority of direct equity investments by SWFs, including unlisted firms, real estate, and infrastructure investments. With listed assets representing less than 20% of total SWF investments, previous analyses offer only a partial – albeit important – view of sustainable investments by SWFs. One important exception is the analysis by Andonov, Kräussl and Rauh (2021) on the determinants of infrastructure investment in closed-end funds. The paper shows that public institutional investors, including SWFs, invest significantly more in infrastructure funds, and that ESG preferences and regulations explain a sizable share of their increased allocation. By considering all asset classes in the equity space, we claim that our paper provides a more comprehensive – albeit mainly descriptive – account of sustainable investing by SWFs.

A few papers have investigated the effect of SWF investment on the subsequent ESG performance of target firms. In an empirical test on the oil and automobile industry, Liang and Renneboog (2020) do not find evidence that SWF ownership increases the ESG performance of the firms belonging to the industries concerned, even if SWFs do select companies with better ESG performance. Chen et al. (2022b) analyze the impact of SWFs' cross-border equity acquisitions on targets' corporate governance and the role the institutional environment of SWF countries plays in shaping this impact. They find that small stakes acquired by SWFs contribute to deteriorating the target firms' corporate governance. This negative impact is stronger for weakly governed firms and those located in jurisdictions with weak shareholder protection. We also contribute to this stream of research by corroborating the view about the negative impact using different ESG performance data.

## THEORETICAL FRAMEWORK

As state-sponsored investors, in principle SWFs are ideally placed to embrace sustainable investment. Due to the sheer size of their assets and long-term investment horizon, SWFs have the potential to catalyze change with regard to eliminating pollution, improving working conditions, pursuing gender equality, and promoting corporate governance (Liang & Renneboog, 2020; Wurster & Schlosser, 2021). However, SWFs are a very heterogeneous group, composed of institutions with different mandates stretching from fiscal revenues stabilization and inter-generational savings to national economic development. Within the category usually labeled strategic investment funds (SIFs), we find many examples of funds with a strong focus on sustainable development by adopting a “double bottom line” approach, targeting financial return and socio-economic impact. A standard setter in this space is the Irish Strategic Investment Fund, but virtually all African SWFs, despite their smaller scale when compared with their global peers, have missions aligned with delivering on SDGs, focusing on sectors such as food and water security, energy generation, healthcare and digitalization, which will have a material impact on their citizens’ lives (IFSWF, 2021). These considerations allow us to state our first theoretical hypothesis.

### Hypothesis 1:

Strategic SWFs, or funds with a developmental mandate should engage more in sustainable investment relative to savings and stabilization funds, typically more oriented towards financial returns.

SWF commitment to sustainable investing could also be affected by political considerations. If one looks at the recent history of SWFs, the largest institutions have successfully built their reputation as purely financial players and assuaged the recipient countries’ concerns that they were pursuing a political agenda in their investments abroad (Kotter & Lel, 2011). This strategy was enshrined in the Santiago Principles, drafted in 2008 to promote transparency, good governance, accountability, and prudent investment practices, and signed by an increasing number of SWFs over the last decade (IFSWF, 2018). At a closer look, SDGs are ultimately global policy goals, and any shift from conventional to sustainable investment would make SWF look more “political”, blurring the boundaries between government activity and sovereign investment that have been laboriously built over the years.^3^ These issues have more practical implications than one might think. They have spiced up the last national elections in Norway, where a debate was ignited over concerns about the political use of the fund in the pursuit of global challenges such as climate change.^4^ SWFs tend to adhere more strictly to their fiduciary duty and avoid venturing out into deals (such as sustainable investments) that would make them appear politically motivated. This risk could be mitigated by governance arrangements that foster a higher degree of managerial independence from the funds’ political sponsors (Bortolotti, Fotak, & Loss, 2019). This leads to our second hypothesis.

### Hypothesis 2:

Politically independent SWFs with a stronger governance framework should engage more in sustainable investment, especially abroad.

The observed sustainable investments can be affected by the presence of an explicit ESG policy at the fund level, as SWFs may or may not state to incorporate ESG considerations in their investment decisions. Indeed, Liang and Renneboog (2021) find that about half of the 24 SWFs included in their study formally disclose their ESG policies in their annual statements, which are related to higher value-weighted ESG ratings of the public equity portion of their portfolios. According to United Nations Conference on Trade and Development (UNCTAD, 2020), out of the 30 largest SWFs, only four funds – i.e., Australian Future Fund, Samruk-Kazyna of Kazakhstan, New Zealand Superannuation Fund (NZSF, New Zealand) and Singapore’s Temasek – report a meaningful ESG integration in their investment strategy. Nevertheless, since the announcement of UN SDGs in 2015 and the global spread of ESG investing, many countries have mobilized their SWFs to support the delivery of these goals, and the number of SWFs with explicit ESG policies has grown significantly (Lopez, 2021). From these observations, we can state our third hypothesis.

### Hypothesis 3:

SWFs with an explicit ESG policy in place should engage more in sustainable investing.

Finally, the literature on state ownership of firms has established that government control is a double-edged sword for the aggregate ESG performance of target companies (Megginson & Fotak, 2015). On the one hand, governments are long-term, patient shareholders, with multi-generational investment horizons, and a greater propensity to finance activities that generate social and environmental returns (Arrow, 1962). More specifically, being SWFs universal owners, namely institutional investors with large, diversified portfolios, they are willing to internalize global externalities such as climate change or income inequality in their investment decisions (Monks & Minnon, 1995). On the other hand, SWF ownership might weaken managerial incentives and corporate governance. SWFs tend to be passive investors, seldom involved in the monitoring of management and therefore failing to provide the traditional benefits of institutional investors (Boubakri, El Ghoul, Guedhami, & Megginson, 2018; Kotter & Lel, 2011). More specifically, Boubakri, Guedhami, Kwok and Wang (2019) have shown that privatized firms tend on average to be more socially responsible than private listed firms. However, a trade-off arises between the sustainability orientation and profit maximization resulting in a nonlinear relationship between state ownership and CSR intensity. Indeed, partially privatized companies under tight government control are more socially responsible than companies with lower residual stakes. As on average SWF tend to acquire minority stakes, we posit that investee companies would not become more socially responsible after the investment.

Aggregate ESG scores lumping together the E, S, and G pillars would not allow capturing the sustainability trade-offs identified above. We thus split them into their individual components, and this leads to our last hypothesis.

### Hypothesis 4a:

The environmental (E) and social (S) performance of SWF targets should not improve post-investment.

### Hypothesis 4b:

The governance (G) performance of SWF targets should deteriorate post-investment.

## DATA

The sample of the SWF investments originates from the SWF Global Transaction Database built by the Sovereign Investment Lab at Bocconi University and comprises 3,565 investment transactions made by 42^5^ tracked SWFs or by their investment vehicles from 2000 to 2020, for a total aggregated deal value of $1.009 trillion. The data include investments in listed and unlisted equity, real estate and real estate funds, private equity and open-ended investment funds, platforms, and joint ventures in which an SWF (either directly or through their financial arms) is an investor. Data collection was performed both with centralized sources (Zephyr, Refinitiv, Preqin, S&P Capital IQ, Bloomberg) and by manually collecting information from publicly available sources such as newspaper articles.

Our sources report equity transactions for 33 of the 42 tracked funds. However, the SWFs included in this analysis represent 96% of the total assets under management of the SIL universe, which is worth USD 6.3 trillion. Furthermore, we have cross-checked the SIL database with the one created by the International Forum of Sovereign Wealth Funds (IFSWFs) including transactions since year 2015. In the period where the two databases overlap (2015–2020), SIL reports 40% of IFSWF deals, accounting for 89% of deal value. From this preliminary analysis, we conclude that the SIL database provides comprehensive and – to the best of our knowledge – unique coverage of large-scale transactions executed by major SWFs over the last two decades. The sustainability dimension pertains to the core business of the investee company: each company reported in the database was manually classified based on its main field of activity according to categories and themes modeled on the IRIS+ taxonomy. Reclassification was based on information contained in the news articles related to the transaction and publicly available information on the investee company at the time of the SWF’s investment.

Each investee company was classified according to categories and themes (“subcategories”) based on the IRIS+ taxonomy (Table 1):

**Table 1:** Deal SDG classification based on IRIS+ taxonomy

IIRIS+ category	IRIS+ theme	Deals	SDG deal value (USD million)
Agriculture	Food Security	9	324
	Smallholder Agriculture	6	127
	Sustainable Agriculture	8	559
Air	Clean Air	–	–
Biodiversity and Ecosystems	Biodiversity and Ecosystem Conservation	24	1,864
Climate	Climate Change Mitigation	18	2,252
	Climate Resilience and Adaptation	–	–
Diversity and Inclusion	Gender Lens	–	–
	Racial Equity	–	–
Education	Access to Quality Education	25	1,823
Employment	Quality Jobs	7	517
Energy	Clean Energy	67	10,017
	Energy Access	4	2,048
	Energy Efficiency	7	1,530
Financial Services	Financial Inclusion	51	2561
Health	Access to Quality Health Care	247	28,601
	Nutrition	2	250
Infrastructure	Resilient Infrastructure	50	18,875
Land	Natural Resources Conservation	–	–
	Sustainable Land Management	–	–
	Sustainable Forestry	1	626
Oceans and Coastal Zones	Marine Resources Conservation and Management	–	–
Pollution	Pollution Prevention	2	60
Real Estate	Affordable Quality Housing	7	308
	Green buildings	4	43
Waste	Waste Management	12	183
Water	Sustainable Water Resources Management	3	147
	Water, Sanitation, and Hygiene (WASH)	10	736
SDG Deals		564	73,449
Non-SDG Deals		2,950	936,260
Missing IRIS+ classification		51	11,734

The category “Non-SDG” was added to the original IRIS+ taxonomy in order to account for investee companies in the database whose core activities did not fit with any of the IRIS+ categories. For 51 companies (for a total deal value of USD 11.8 billion) it was not possible to find information to characterize their sustainability profile, and therefore we treat them separately as cases with missing data. If more than one IRIS category could be applied to a certain investee company, the one which fitted the largest share of the investee company’s core activities best (according to the information found) was adopted.

A few caveats are in order. First, the focus of this report is SWF investments with a sustainability profile. This subset of transactions presents a very skewed distribution with respect to deal value: ten investee companies account for more than 47% of total sustainable deal value recorded in the database. This may lead in some instances to incorrect conclusions, because the significant weight of certain categories would be driven not by a robust underlying trend, but instead by few large deals. As a remedy, for most of the analyses presented in this report, the “winsorization” method popular in economic and financial literature was applied to the variable “deal value”: the top and bottom 1% of the data was removed in order to exclude the largest and smallest outliers. In the top 1% of sustainable deals, four transactions were excluded: Qatar Railways Development Company (18% of total sustainable deal value), Bayer AG (5%), Nestle Skin Health SA (4.6%) and Allergan Plc (4%).

The sustainability classification of investee companies was performed following the detailed guidelines of IRIS+. Being a manual classification based on publicly available sources, a certain degree of subjectivity in the classification process was unavoidable. Some examples of borderline cases are:

- Nuclear energy and natural gas: companies operating in these fields were not considered sustainable investments despite the important role they play in the transition from oil and carbon to more renewable sources of energy (natural gas is still a fossil fuel and nuclear energy is in many countries not socially accepted);
- Telecom investments are generally considered sustainable investments; we tried to find out if the investment was done in underserved communities even if the definition of an underserved community might be challenging. In many emerging markets, telecom investments have generally led to vastly positive developmental outcomes in terms of financial inclusion, mobile money applications, jobs, and generally beneficial innovation;
- “Social innovation” companies (those who promise to change the life of millions with artificial intelligence and the like) were not considered sustainable investments per se, unless there was a clear and proven connection with one of the IRIS+ categories.

Out of the total 3,565 transactions recorded in our database, we were able to flag 564 transactions representing a total of USD 73.5 billion of aggregated transaction value as ‘sustainable (SDG) investments’. This represented 16% of the total deal count and 7% of the total deal value of the SWF transactions. The distribution of deal value within SDG investments is skewed (even after performing winsorization, as described in the previous section) but this is simply a function of the highly heterogeneous nature of the SWF community, with a considerable diversity in mandates, as mentioned in the introduction (Table 2).

**Table 2:** Summary statistics (winsorized data)

	Deals	Deal value (USD million)	# Available deal values	Average deal value (USD million)	Standard deviation (USD million)	Min. deal value (USD million)	1st Quartile (USD million)	Median (USD million)	3rd Quartile (USD million)	Max deal value (USD million)
All deals	3,557	998,124	2,625	380	1,356	0	20	89	300	45,000
SDG deals	56	50,131	436	115	276	1.04	10	31	90	2,639
Non-SDG deals	3,001	947,993	2,189	433	1,475	0	25	105	350	45,000

Given a possible subjectivity in IRIS+ classification, we try to test how our taxonomy compares with conventional ESG scores.

As recently documented by Berg, Kölbel and Rigobon (2022), the correlation among prominent agencies’ ESG ratings is on average quite low, and this ambiguity around the consistency of ratings has created acute challenges for investors and researchers alike. A new approach has been taken by RepRisk, a leading ESG data vendor translating big data into ESG risk metrics using artificial intelligence and machine learning. RepRisk systematically screens daily news over 100,000 public sources in 23 languages, including newspapers, social media, government bodies, regulators, and other public sources. The RepRisk ESG Risk Platform identifies adverse ESG incidents and evaluates material ESG risks for 180,000+ public and private companies and 45,000+ infrastructure projects on a rule basis. RepRisk’s research scope covers 28 ESG issues under international standards, namely the World Bank Group Environmental, Health, and Safety (EHS) Guidelines, the IFC Performance Standards, the Equator Principles, and the OECD Guidelines for Multinational Enterprises. RepRisk also maps its data into international ESG and regulatory frameworks, such as 17 SDGs, ten principles of the UN Global Compact, the SASB Materiality Map, and others. The aggregate RepRisk score and its sub-scores on E, S, and G have been published on a monthly basis since 2007. The score for each target firm at each recorded point in time ranges from 0 (no risk) to 100 (maximum risk). For each value of the score, the relative weight of each of the sub-components E, S, G is provided. We employed for the analysis the total RepRisk score and estimated a score for each of the individual components simply by multiplying the total RepRisk score for each of the weights of the components E, S, G, respectively.

We found 255 of the 2,989 target companies included in our database in the RepRisk platform. Thirty (12%) are classified as SDG investments, and they are all listed companies. Table 3 reports the average RepRisk scores in the 17 IRIS+ sectors and some descriptive statistics. Health is by far the sector which accounts for the largest number of deals and exhibits the second lowest average value for the RepRisk score (9.5) among the IRIS+ categories listed. Infrastructure and Energy follow suit on a lower scale, with seven and five deals respectively and an average RepRisk score of 13.7 and 9.9, respectively.

**Table 3:** Target companies’ SDG classification and ESG scores

SDG (IRIS+) categories	Observations	RepRisk Index	RepRisk Index (E)	RepRisk Index (S)	RepRisk Index (G)
Agriculture	1	21.96	9.34	8.25	4.37
Biodiversity and Ecosystems	1	2.28	0.00	2.28	0.00
Energy	5	9.86	3.56	4.29	2.01
Health	14	9.55	2.22	2.97	4.36
Infrastructure	7	13.68	1.08	4.43	8.16
Water	2	10.06	3.70	3.90	2.46
SDGs (means)	30	10.77	2.44	3.75	4.58
Non-SDG (mean)	225	11.80	2.51	4.11	5.18
SDGs (median)	30	6.59	0.47	2.22	2.11
Non-SDG (median)	225	7.21	0.75	2.55	2.85
Total (mean)	255	11.68	2.50	4.07	5.11
Total (median)	255	7.15	0.74	2.41	2.84

The mean and median RepRisk scores are always lower in the SDG than in the non-SDG sample, suggesting that the companies that we classified as sustainable also have lower aggregate ESG risk. Interestingly, the difference between the medians of the environmental score E is particularly pronounced, indicating that our SDG deals rank particularly high in terms of environmental sustainability. We performed two-tailed *t* tests for the difference in means between the sample of SDG and Non-SDG investments alongside Mood’s tests for the difference in medians: although the signs of the statistics indicate a lower risk associated with SDG investments, we found the results to be not statistically significant.^6^ This comparison based on the subsample of listed firms suggests that the SDG flag based on the IRIS+ taxonomy can be considered a proxy for a high ESG score in terms of lower risk. We tentatively assume that the same association between SDG flags and ESG risk could also hold for unlisted companies, so far under the radar screen of ESG rating providers.

We have computed the total number of transactions at the fund level, SDG DEALS, and the corresponding total USD amount, SDG VALUE. We have then collected fund-specific information from Global SWF, a data platform for SWF and state-sponsored pension funds,^7^ the IFSWF, and from the Sovereign Investment Lab. These sources consistently classify SWF according to their primary mission: savings, pension, and development funds. We therefore flag funds with a development mandate as STRATEGIC, and use this dummy variable in the empirical test of H1.

Global SWFs publishes the GSR Scoreboard, a tool to analyze governance, sustainability, and resilience organizational efforts by SWFs.^8^ The scorecard raises 25 questions: ten related to Governance, Transparency and Accountability; ten concerning Sustainability and Responsible Investing; and five on Resilience and Legitimacy. These questions are answered binarily (Yes/No) with equal weight and the results are converted into a percentage scale for each of the funds. We are particularly interested in the GOVERNANCE, and SUSTAINABILITY sub-scores, which allow us to identify funds with more solid governance and stronger managerial independence, and those with full-fledged ESG policies and risk management frameworks in place. These variables will be used in the empirical test of H2 and H3, respectively^9^.

We will also use as additional variables the dollar value of individual SWFs’ assets under management, AUM, to control for size effects in the scale of SWFs’ sustainable investment programs, and an indicator about the sources of funding, COMMODITY, to test any different behavior by SWFs from oil exporting countries in embracing SDGs. Table 4 presents the key data and indicators at the fund level that will be used in the econometric analysis.

**Table 4:** SWFs’ sustainable investment

SWF	Country	Deals	Deal value (USD million)	SDG deals	SDG deal value (USD million)	Funding	Mission	AUM (USD billion)	GSR score	Governance	Sustainability
Abu Dhabi Investment Authority	UAE	181	55,111	20	6,592	Commodity	Savings	726	52	6	4
ADQ Holding Company PJSC	UAE	17	2,184	5	–	Commodity	Savings	–	–	–	–
Mumtalakat Holding Company	Bahrain	16	414	5	215	Commodity	Development	19	44	7	5
Brunei Investment Agency	Brunei	25	1,117	1	–	Commodity	Savings	45	4	2	–
China Investment Corporation	China	278	176,744	21	3765	Non-Commodity	Savings	1,046	60	9	5
Dubai International Financial Centre	UAE	14	2,867	–	–	Commodity	Savings	–	–	–	–
Emirates Investment Authority	UAE	4	6,824	–	–	Commodity	Development	63	12	3	2
Fundo Soberano de Angola	Angola	4	180	–	–	Commodity	Development	5	24	10	5
Future Fund	Australia	57	7,843	10	632	Non-Commodity	Savings	150	100	10	9
Government Investment Corporation	Singapore	732	171,800	88	14,578	Non-Commodity	Savings	488	60	6	5
Government Pension Fund-Global	Norway	65	18,281	4	18	Commodity	Savings	1,128	96	9	8
Investment Corporation of Dubai	UAE	22	7,130	2	103	Commodity	Development	305	28	7	4
Ireland Strategic Investment Fund	Ireland	56	14,552	19	428	Non-Commodity	Development	12	80	9	10
Istithmar	Morocco	2	250	–	–	Non-Commodity	Development	13	16	7	10
Kazakhstan National Fund	Kazakhstan	3	406	–	–	Commodity	Stabilization	62	56	7	10
Khazanah Nasional Bhd	Malaysia	109	15,831	33	2236	Non-Commodity	Development	33	48	5	8
Korea Investment Corporation	South Korea	35	4,156	3	61	Non-Commodity	Savings	157	60	8	8
Kuwait Investment Authority	Kuwait	122	31,621	10	492	Commodity	Savings	559	36	5	5
Libyan Investment Authority	Libya	53	5,470	6	222	Commodity	Savings	67	4	7	5
Mubadala Investment Company	UAE	343	131,139	51	5,974	Commodity	Development	232	72	9	8
National Social Security Fund	China	40	12,952	–	–	Non-Commodity	Pension	376	32	6	2
National Wealth Fund	Russia	2	2,472	–	–	Commodity	Stabilization	174	20	3	1
New Zealand Superannuation Fund	New Zealand	48	3,163	12	1,334	Non-Commodity	Savings	31	96	10	9
Oman Investment Authority	Oman	85	5,014	9	405	Commodity	Development	31	40	2	4
Palestine Investment Fund	Palestine	3	60	2	60	Non-Commodity	Development	1	48	6	6
Public Investment Fund	Saudi Arabia	42	59,612	8	1139	Commodity	Development	360	28	5	8
Qatar Investment Authority	Qatar	290	151,463	29	21,533	Commodity	Savings	345	32	5	7
RAK Investment Authority	UAE	4	12	–	–	Commodity	Savings	–	–	–	–
Russian Direct Investment Fund	Russia	50	2,042	8	13	Non-Commodity	Development	31	48	3	4
State Capital Investment Corporation	Vietnam	3	95	-	-	Non-Commodity	Development	1	24	5	4
State Oil Fund of Azerbaijan	Azerbaijan	34	3,381	3	244	Commodity	Stabilization	43	52	10	6
Temasek Holdings	Singapore	830	116,287	218	13,414	Non-Commodity	Development	215	92	9	10
Turkey Wealth Fund	Turkey	11	11,325	–	–	Non-Commodity	Development	34	48	6	5

## DESCRIPTIVE ANALYSIS

Perhaps not surprisingly, we do see an increasing appetite by SWFs to make sustainable investments over time. The growth in SDG deal count from 2010 onward may well have benefitted from the spike in overall deal-making, both in deal count and deal value. In the aftermath of the Global Financial Crisis (GFC), several SWFs made large value-driven investments in alternative assets such as private equity and real estate. After reaching a plateau of around 27 deals a year in the 2012–2016 timeframe, we see the total sustainable deal count (the total number of SDG deals) accelerate to 59 in 2017 and progress strongly to almost 100 in 2020.

**Figure 1:**
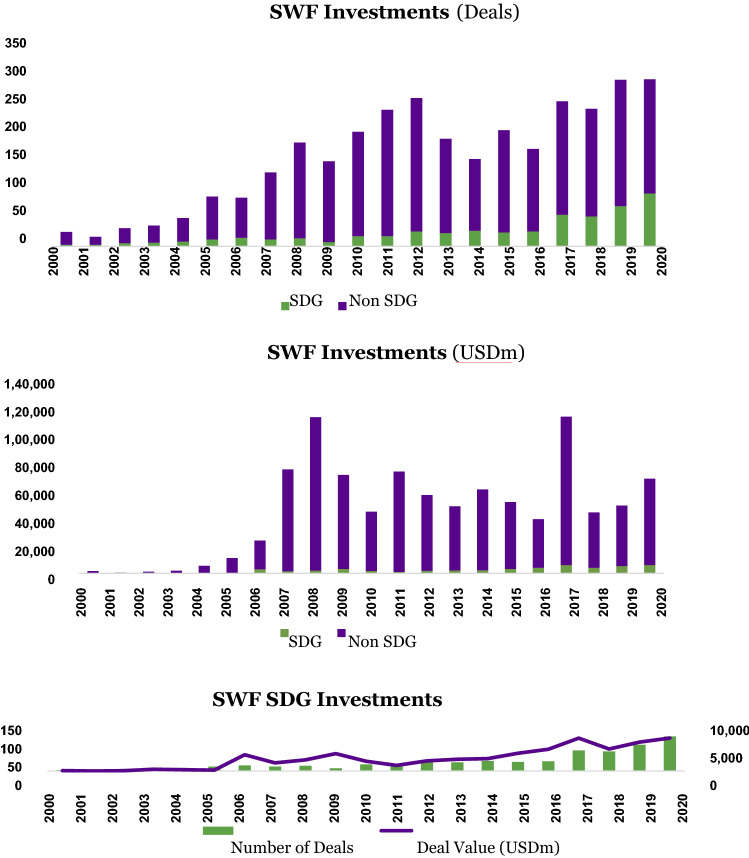
SWF investments by deal count and value

We hypothesize that the considerable uptick in 2017 from a low base and the subsequent steady increase from that year onwards in the deal count come from two different but interlinked catalytic global milestones, each in 2015: the adoption by the 193 UN member states of the SDGs themselves in September 2015 and the adoption by 196 parties of the Paris Agreement, a legally binding international treaty on climate change, in December 2015. Institutional investments are carefully planned and considered well ahead of time and closing them can take up to a year. It is therefore plausible that after a certain time lag, during most of 2016, some of the largest and more progressive SWFs began to be sensitized to the importance and urgency of the new global policy frameworks as well as the opportunities afforded by them, and consequently started changing their screening, investment and decision-making processes to align with these new realities (Figure 1).

The total sustainable deal value (the total USD amount of SDG investments) is also keeping pace in these later years. We see a spike in total sustainable deal value in 2017 to around USD 6.3 billion, a brief dip in 2018, and then a steady increase by 2020 back to 2017 levels.

### Sectoral Analysis

**Figure 2:**
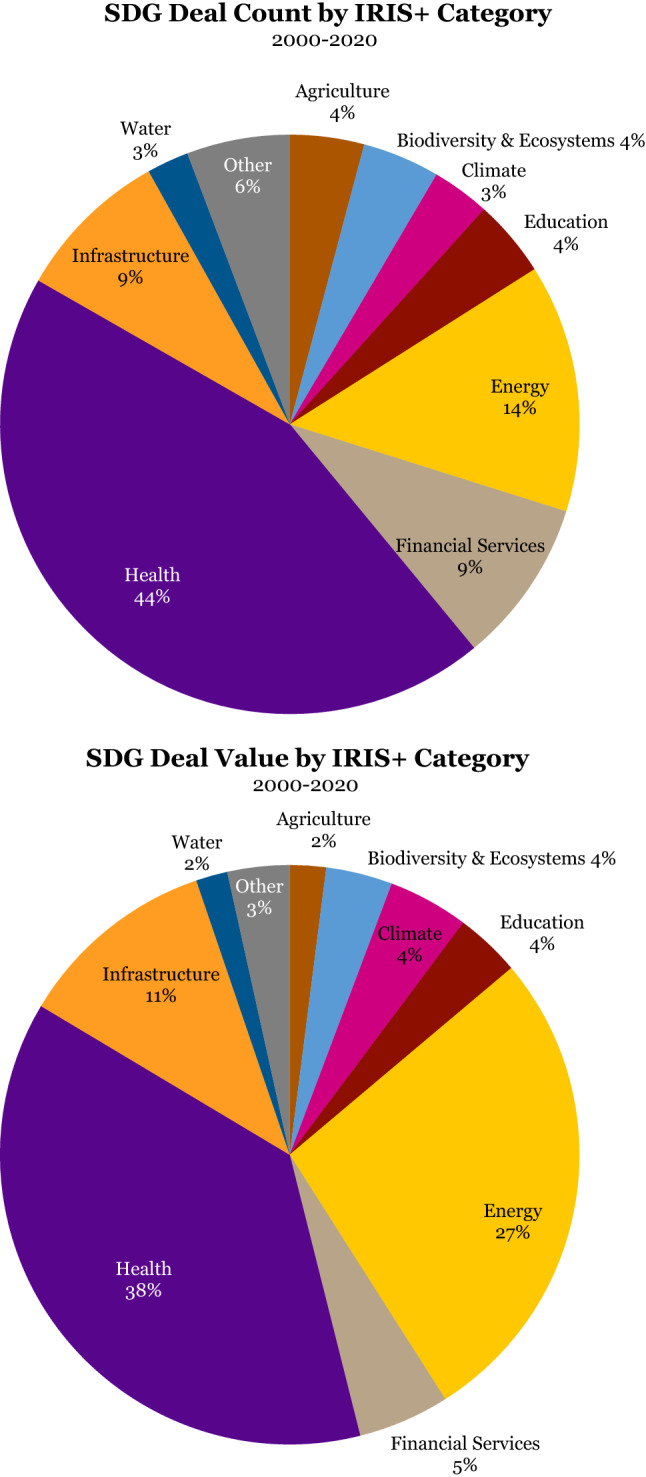
SDG deal count and value by IRIS+ category

When we perform a sectoral analysis across the two decades covered by the database, what immediately stands out is that SWFs’ sustainable investments are predominantly in the sectors of healthcare, energy, financial services, and infrastructure. The healthcare sector makes up 44% of the sustainable deal count and 38% of sustainable deal value, making it the leading sector by some distance (Figure 2)^10^.

**Figure 3:**
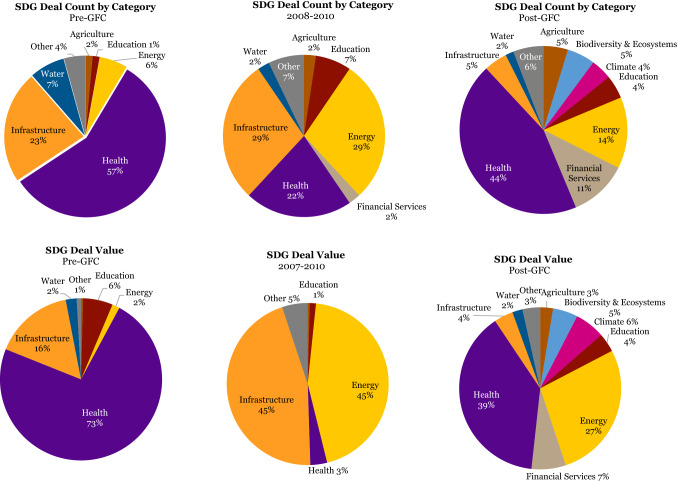
SDG deal count and value by IRIS+ category before and after GFC

Energy follows, representing 14% of the SDG deal count and 27% of the deal value, respectively. Financial services and infrastructure vie for third and fourth place, with both 9% in SDG deal count, and 5% and 11% in sustainable deal value. Energy, financial services, and infrastructure are all considered sectors with highly investable business and revenue models that are generally well tested and understood. Other more niche or nascent investment sectors, with more challenging business and revenue models for mainstream institutional investors, including water, education, agriculture, climate, and biodiversity and ecosystems, individually represent 4% or less (Figure 3).

Looking at the proportion of the four leading investment sectors in sustainable deals across three different time spans – the pre-GFC years, the GFC itself from 2008 to 2010, and the post-GFC era – some interesting shifts through the two decades become visible.

Pre-GFC the healthcare sector was even more dominant in terms of sustainable deal value, at a whopping 73%, and sustainable deal count at 57%. Infrastructure followed, with 16 and 23% respectively. However, we should caution that these early years are statistically not very meaningful due to the low SWF activity in sustainable investments in general.

During the GFC SWFs shifted their sustainable investments significantly into infrastructure (45% of deal value, and 29% of deal count) and energy (45% of total deal value and 29% of deal count). The healthcare sector bore the brunt of the re-direction of investment flows and fell to just 3% in sustainable deal value and 22% in deal count during this period. Sustainable financial services received scant attention during these same years as most investment in the industry was targeted toward the rescue of US battered banks.

The probable cause of this shift during the GFC may well have been that in times of severe economic stress, SWFs, by their own design or by the request of their political masters, elect to commit capital to capital-intensive hard assets like infrastructure and energy projects that stimulate the economy and create employment. This allows investors to enjoy predictable long-term cash flows over a long investment horizon.

**Figure 4:**
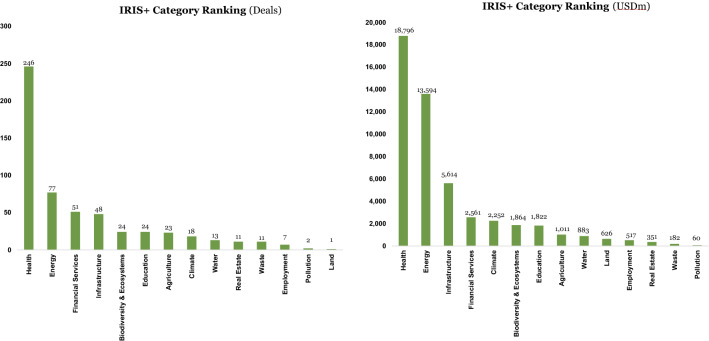
IRIS+ category ranking by deal count and value

What happened next may lend credibility to this possible explanation. After the GFC, investment in the infrastructure sector fell precipitously from 45 to just 4% in sustainable deal value, and from 29 to 5% in sustainable deal count, even below pre-GFC levels. Energy experienced a less hefty decline, from 45 to 27% in sustainable deal value, and from 29 to 14% in sustainable deal count. It is plausible that after the investment spike during the GFC in infrastructure projects, the number of available ‘spade-ready’ infrastructure projects had been exhausted. Energy projects specifically, however, have a longer design, engineering, procurement, and construction phase, and hence the ‘rise and fall’ effect may be less pronounced (Figure 4).

The graphs above confirm the leading investment sectors, both by deal count and deal value: overall healthcare is by far the biggest beneficiary of SWFs’ sustainable investments, followed by energy, financial services, and infrastructure.

### Geographic Analysis

Changing our vantage point to a geographic analysis of sustainable investments made by SWFs over the two decades covered, Europe and North America received the largest SDG deal value, with 26 and 25% respectively, followed by Asia–Pacific with 20%, and Southern Asia with 18%. Middle East, Latin America, and Africa trail with a modest 7, 3, and 1%, respectively.

Interestingly, the deal count shows a slightly different distribution. Here, the lead goes to North America and Southern Asia, both with 24%, followed by Asia–Pacific with 21%, and Europe with 18%. Middle East and Africa with a combined 10% and Non-Pacific Asia together with Latin America (with 2 and 1%, respectively) close the ranks.

This pattern is not surprising though. Europe and North America are lower-risk OECD economies with many significant and mature public and privately held companies. Europe may well be receiving the largest dollar share of sustainable investments since it is more dialed into sustainability than North America.

**Figure 5:**
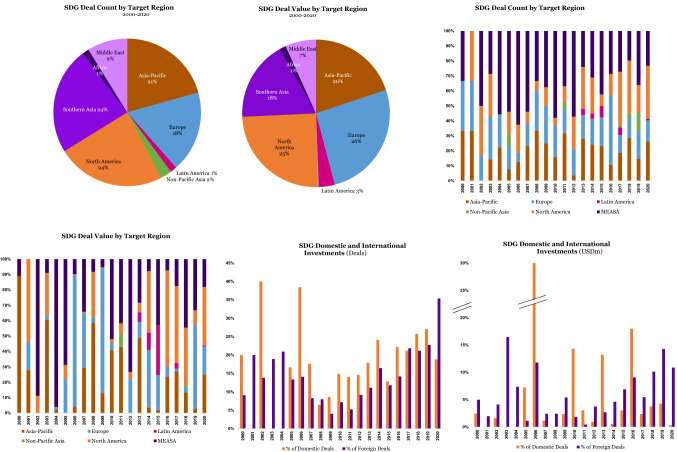
SDG deal count and value by target region

MEASA (the Middle East, Africa, and Southern Asia) gets the larger share of deal value (26%), and deal count (34%) due to the contribution of Southern Asia (particularly driven by India and Singapore), while the other two sub-regions fall behind, and spectacularly so. MEASA combines the highest long-term growth potential, thanks to the demographic dividends of Southern Asia, with the most acute socio-economic problems of the less developed nations. We claim that transition investments in the MEASA region by international and domestic SWFs will be a key challenge in the years to come (Figure 5).

Regional data do not show a lot of variances from the overall trends, and hence we leave it unreported here. The healthcare sector shows the largest sustainable deal count in all target regions except for Africa, where energy and infrastructure play the biggest role as investment sectors – this is to be expected in a continent where electrification is still the single largest development need – followed by investments in agriculture and real estate.

When it comes to sustainable deal value, we see the healthcare sector again take the lion’s share in Europe (aging population), Latin America (rising middle classes, and fragile healthcare systems), Middle East (rising middle classes, catered to by nascent healthcare systems), and North America (aging population, and healthcare represents a disproportional 19.7% of GDP in 2020^11^). In the Asia–Pacific region too, healthcare takes the runner-up position, after energy and infrastructure in the leading positions, in a region that sees its energy needs double in the next decade due to high economic growth projections.

A final noteworthy geographical perspective is to be found in the two graphs above which report the share of SDG investments in domestic vs. foreign deals. Interestingly, deal count, and deal value tell two different stories. By deal count, SWFs seem more prone to make SDG investments at home, which is consistent with a mandate of socio-economic development. By deal value, however, we see the opposite: in most years, and markedly since 2017, the share of SDG investments abroad has outpaced the share made at home. By taking a closer look at transaction data across deal types, we discover that domestic SDG investments are significantly smaller than international ones. Indeed, the mean (median) size of domestic SDG deals is USD 54 (21) million, while it rises to USD 100 (34) for SDG investment abroad. A working hypothesis is that this may be attributable to a “firm size” effect. While chasing SDG opportunities at home, SWFs are willing to take an early stage venture capital approach, by investing in innovative startups with a high potential to generate spillover effects in the local economy. When they invest abroad, SWF pick instead larger, more established, and consequently less risky companies, seeking primarily financial returns rather than impact.

### Funds

**Figure 6:**
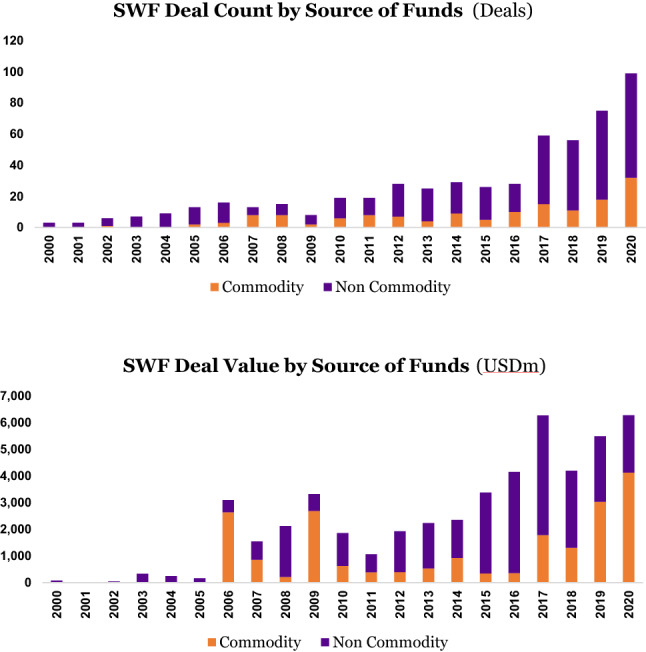
SWF deals by source of funds

Another interesting question is whether there is a relationship between the degree to which a SWF makes sustainable investments and the source of a SWF’s funds, whether its origin be commodity-related wealth or non-commodity related wealth? (Figure 6).

**Figure 7:**
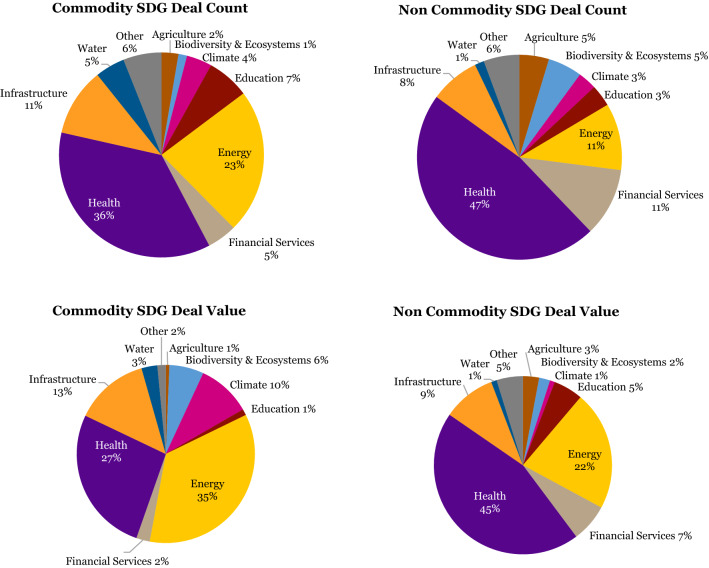
SDG deals by source of funds

The above graph shows that over the past 5 years, both commodity SWFs and non-commodity SWFs have begun to make more sustainable investments, both in terms of deal count and deal value (Figure 7).

In terms of sectoral investment focus, the two types of funds behave largely the same, with a strong focus generally on the healthcare, energy, infrastructure, and climate sectors, albeit that the non-commodity SWFs relatively invest more in healthcare (45% of deal value) and relatively less in energy (22%) compared to their commodity peers (27% for healthcare, and 35% for energy). The larger value of investments made by the commodity SWFs in energy and climate (together representing 44% of deal value), relative to their non-commodity peers (23%), would suggest there is a focus on diversifying their portfolios into clean and renewable energy away from the hydrocarbons that predominantly form the source of their wealth.

When we disaggregate the data further down to the level of individual SWFs, we see some sustainable investing champions in the field emerge (see “Appendix”).

The two generally forward-looking Singaporean SWFs, GIC Pte Ltd. (GIC) and Temasek Holdings Inc. (Temasek) lead the ranking with USD 11.6 billion (7% of their total deal value), spread over 86 deals (12% of their total deal count), and USD 9.7 billion (9% of deal value), distributed over 213 deals (26% of their deal count), respectively.

There are other noteworthy players in this echelon. Qatar Investment Authority invested more than USD 8 billion (6% of total deal value) in SDG investments spread over 28 deals (10% of deal count), whereas Mubadala Investment Corporation PJSC invested almost USD 6 billion (5% of total deal value) across 50 deals (15% of total deal count). China Investment Corporation (CIC) invested USD 3.8 billion (2% of total deal value) across 21 deals (8% of total deal count), and Abu Dhabi Investment Authority (ADIA) is not far behind, with USD 3.2 billion in SDG deals (6% of total deal value) invested across 19 deals (11% of deal count). The league tables would thus be led by the largest Middle Eastern SWFs, the Singaporean, and Chinese SWFs.

In the next echelon, we find three more SWFs investing at least USD 1 billion in the aggregate in SDG deals over the period. Khazanah Nasional Bhd of Malaysia invested USD 2.2 billion (14% of total deal value) across 33 deals (30% of deal count). The New Zealand Superannuation fund represented USD 1.3 billion of investments (45% of deal value) in 12 deals (26% of their deal count), and, last but not least in this category, the quickly growing Saudi Public Investment Fund (PIF) invested USD 1.1 billion (2% of total deal value) in eight deals (20% of total deal count).

**Figure 8:**
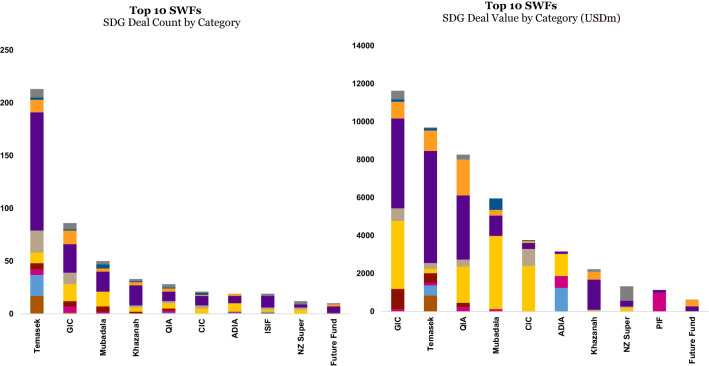
Top 10 SWFs – SDG deals

Another interesting way of looking at this data is the prevalence of sustainable investing as applied to investment decisions (expressed as a percentage of deal value and deal count) in some select SWFs, suggesting a more consistent integration of sustainability considerations in their investment operations. For example, 52% of Bahrain Mumtalakat Holding Company’s deal value was sustainability-driven, representing a total of USD 215 million across five investments. The New Zealand Superannuation Fund, which, as highlighted earlier, has developed a sophisticated strategy against climate change, had a sustainability-driven deal value of 45%, representing USD 1.3 billion invested in 12 deals (Figure 8).

Finally, taking a closer look at the target sectors that the top five sustainability-driven SWFs – Singapore’s Government Investment Corporation and Temasek, Qatar Investment Authority, Mubadala and China Investment Corporation – invested in, the healthcare sector is the predominant target of investments, in terms of deal count (ranging from 31 to 53%), for all five, invariably followed by a combination of energy, financial services and infrastructure. All four sectors taken together typically make up around three quarters of the total deal count.

The deal value analysis largely reinforces, with some exceptions, the dominance of the healthcare as a target investment sector, making up 41% of deal value (or USD 4.7 billion) for GIC, 41% (or USD 3.4 billion) for QIA and a whopping 61% (or USD 5.9 billion) for Temasek. This is also consistent with the league table of sustainable investments where healthcare dominates the largest six investments made (not taking into account the winsorization): Temasek invested $3.7 billion in Bayer AG in 2018, and USD 1.8 billion in US healthcare firm Global Healthcare Exchange in 2017, while GIC invested nearly USD 3 billion in Allergan of Ireland in 2017 and USD 2.5 billion Multiplan of the US in 2016. Meanwhile, QIA invested USD 2.6 billion in British firm Four Seasons Healthcare.

Healthcare is not always the predominant sector: 65% (or USD 3.9 billion) of Mubadala’s deal value was driven by the energy sector, with a notable investment in 2009 of almost USD 1 billion in London Array, an offshore wind farm. The other exception to the dominance of healthcare in terms of deal value was CIC. It invested 64% of its total deal value (or USD 2.4 billion) in energy, led by a USD 1.2 billion investment in Singapore-based Equis Energy Developments Pte Ltd. in 2018, and a relatively modest 8% (or USD 316 million) in nine smaller healthcare deals. The Chinese SWF orientation toward energy may come from Asia’s concern about continuous energy security against the backdrop of fast-growing economies and the associated voracious energy demand.

## EMPIRICAL ANALYSIS

In this section, we present the results of a preliminary empirical test of the theoretical hypotheses stated in the "THEORETICAL FRAMEWORK" section. Some caveats are in order. We do not have available company-specific data for most target firms of SWF investments, our sample being mainly composed of international unlisted firms for which comprehensive balance sheet data are not available. Given this data limitation, we have carried out a cross-sectional empirical analysis of the determinants of SDG investments using the 33 SWFs as the unit of analysis. The small sample size and the limited availability of control variables at the fund affect the explanatory power of our tests. Given this possibility, we admit that we are estimating conditional expectations, and suggest caution to the reader in the causal interpretation of our reported coefficients. Indeed, the reported regression results do not provide conclusive evidence about the hypotheses stated in the theoretical framework.

Table 5 presents the results of our regression analyses where the dependent variable is the total number of SDG deals. In all estimated models, we control for size effects by including the dollar value of an individual fund’s asset under management. Clearly, larger funds have more investment capacity, and this could simply influence the extent of their investment programs in line with the SDGs.

**Table 5:** SWF SDG deals: OLS regressions

Variables	(1)	(2)	(3)	(4)	(5)	(6)
	Dependent variable: SDG deals				Dependent variable: foreign SDG deals	
Intercept	3.33	− 11.43	− 5.37	− 14.04	− 9.45	− 3.81
	(− 15.27)	(14.92)	(22.57)	(18.85)	(13.05)	(19.56)
AUM	0.03	0.00	0.01	0.02	0.00	0.01
	(0.03)	(0.03)	(0.03)	(0.03)	(0.02)	(0.02)
Strategic	17.71					
	(17.43)					
GSR		0.64**			0.51**	
		(0.28)			(0.25)	
Governance			3.32			2.50
			(3.27)			(2.83)
Sustainability				4.92*		
				(2.74)		
Adj *R*^2^	0.022	0.108	0.022	0.052	0.082	0.031
Nobs	30	30	30	30	30	30

Standard errors are reported in parenthesis

***, **, and *represent 1%, 5%, and 10% statistical significance levels, respectively

Our empirical results suggest that stated SWF’s mission is not a critical driver of sustainable investing. The coefficient of the dummy variable STRATEGIC, flagging funds with an explicit developmental mandate and a concentration of domestic assets, is positive but never statistically significant. Indeed, in the same category we find extremely active funds such as Singapore's Temasek and Ireland’s ISIF, and SWFs that are exclusively engaged in conventional, non-SDG investing. Indeed, six out of the ten funds that do not report a single SDG deal are classified as strategic.

We find instead a strong and statistically significant relation between the SDG deals and the aggregate GSR score, a measure of the overall transparency, accountability, and governance of the SWF. This result suggests that the institutional and organizational structure of the fund can play a role in the execution of a more consistent program of SDG deals. However, the GSR score lumps together all contributing factors to a broadly defined SWF accountability across multiple dimensions. In order to run a more rigorous test of our theoretical hypotheses, we will use the GSR score’s individual pillars.

The GOVERNANCE score is a dependable proxy of the actual SWF governance, also including information about the degree of independence granted to fund managers with respect to their political leadership. We do not find, however, any robust relation between SWF governance and SDG investing. The same variable is used as a regressor in the estimation of the number of foreign SDG deals, that allows for a more precise test of H2. According to our theoretical framework, “politicized” funds should be more constrained to pursue sustainable development abroad with respect to funds with a more solid governance framework shielding them from political interference. The estimated coefficient in both models (3 and 6) with different dependent variables is positive, but never significant.

We try to bring to the data our hypothesis H3 on the role of full-fledged ESG policies in place, measured by the SUSTAINABILITY pillar of the GSR score. The estimated coefficient of the variable is economically and statistically significant at conventional levels. A standard deviation increase in the ESG score seems associated with on average 17 additional SDG investments. SWF stating explicitly their ESG considerations and putting in place solid ESG risk management frameworks are more engaged in sustainable investing, at home and abroad.

To summarize, the empirical results shown so far do not seem consistent with H1 and H2, the mission and governance propositions, while seem broadly in line with H3, identifying the presence of ESG policies in place as a predictor of a more intense SDG deal making.

Deal counts are certainly valuable indicators of the extent of SWFs’ sustainable investment programs, but a more comprehensive picture is provided by the actual dollar value of these investments. The variable SDG VALUE captures the actual scale of these transactions, which is obviously determined by the dry powder SWFs have available for investments. In Table 6, we present the results of our regressions where the dependent variable is SDG VALUE suitably scaled by individual SWF’s AUM.

**Table 6:** SWF SDG deal value: OLS regressions

Variables	(1)	(2)	(3)	(4)	(5)	(6)
	Dependent variable: SDG value/AUM				Dependent variable: foreign SDG value/AUM	
Intercept	− 0.42	5.36	− 6.07	5.76	− 1.99	2.00
	(8.00)	(11.78)	(9.10)	(6.52)	(5.60)	(8.31)
Strategic				6.13		
				(8.07)		
Source type	8.67	13.00	8.81	12.69	4.16	7.42
	(8.45)	(8.27)	(7.97)	(8.09)	(5.92)	(5.83)
GSR	0.24				0.18	
	(0.16)				(0.11)	
Governance		0.51				0.47
		(1.72)				(1.21)
Sustainability			2.83*			
			(1.46)			
Adj *R*^2^	0.100	0.029	0.145		0.087	0.002
Nobs	30	30	30		30	30

Standard errors are reported in parenthesis

***, **, and * represent 1%, 5%, and 10% statistical significance levels respectively

For the sake of symmetry, we run the same models of Table 5, adding the dummy COMMODITY as control variable. The estimated coefficients for the (scaled) value of SWG investments presented in Table 6 are consistent with the analysis on the number of deals. Again, we do not find any systematic evidence supporting SWF mission and governance. The coefficient of the aggregate GSR score still confirmed positive, even if not statistically significant. The SUSTAINABILITY sub-score is again positive and significant at conventional level, corroborating previous evidence about the role of ESG policies as a driver of SDG investments by funds.

As a final empirical test, we carry out a preliminary analysis on the effects of SWF investment on the ESG performance of target firms.^12^ As we mentioned in the "THEORETICAL FRAMEWORK" section, the SWF as shareholder can impact the ESG behavior of portfolio companies in several conflicting ways. On the one hand, state ownership and control may provide long-term stewardship towards sustainability goals and improve ESG performance in the environmental and social dimension. On the other hand, SWFs as shareholders can negatively impact the corporate governance of investee firms, exacerbating agency costs and managerial slack by failing to provide the benefits associated with monitoring and strategic oversight by institutional investors. Solving these conflicting views is primarily an empirical issue that previous analyses have tried to address. Liang and Renneboog (2020) could not find any systematic impact on the aggregate ESG ratings of invested firms. However, as already pointed out, these measures lump together different dimensions of the sustainability footprint of firms whereas channels and outcome may differ substantially across themes. Indeed, Chen et al. (2022a) have documented that SWF minority ownership is material in the deterioration of the corporate governance of investee firms, suggesting passivity as a possible channel.

The granular data from our source RepRisk allows us to precisely track the evolution of ESG performance at the aggregate level and across E (environmental), S (social), and G (governance) pillars. We thus compute the difference between the ESG performance before and after the investment by SWFs using different time windows, computing averages for 1, 2, and 3 years pre/post acquisition for the subsamples of SDG and non-SDG firms.

**Figure 9:**
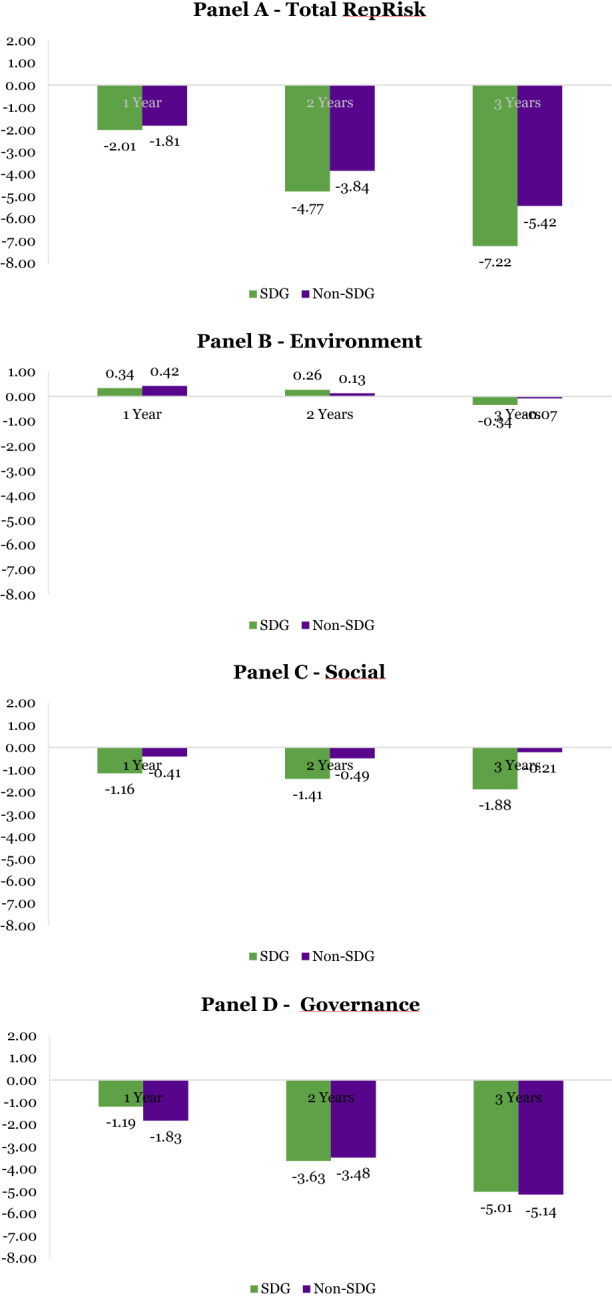
Pre/post SWF investment difference in ESG/RepRisk scores

Results are presented in Table 7 and summarized in Figure 9. We report a progressive deterioration of total ESG performance, which tends to consolidate in absolute value and statistical significance in the longer time windows. The difference in means is largest and most significantly different from zero in the 3 years’ time window before and after the acquisition and slightly more pronounced in the SDG subsample of target firms.

**Table 7:** SWF target firms ESG performance before and after the investment

Panel A: Total RepRisk score						
RepRisk Index	Sample	Average before investment	Average after investment	Difference	Observations	*p* value
Total RepRisk (1 year)	SDG	13.12	15.13	− 2.01	39	0.50
Total RepRisk (2 years)	SDG	12.65	17.42	− 4.77	27	0.17
Total RepRisk (3 years)	SDG	8.83	16.05	− 7.22**	24	0.01
Total RepRisk (1 year)	Non-SDG	15.62	17.43	− 1.81	284	0.20
Total RepRisk (2 years)	Non-SDG	14.55	18.40	− 3.84**	253	0.01
Total RepRisk (3 years)	Non-SDG	14.32	19.75	− 5.42***	211	0.00

Panel B: RepRisk score: Environment						
RepRisk Index	Sample	Average before investment	Average after investment	Difference	Observations	*p* value
RepRisk Environment (1 year)	SDG	4.06	3.72	0.34	39	0.77
RepRisk Environment (2 years)	SDG	4.61	4.35	0.26	27	0.85
RepRisk Environment (3 years)	SDG	3.17	3.50	− 0.34	24	0.77
RepRisk Environment (1 year)	Non-SDG	3.91	3.49	0.42	284	0.41
RepRisk Environment (2 years)	Non-SDG	3.57	3.45	0.13	253	0.80
RepRisk Environment (3 years)	Non-SDG	3.54	3.61	− 0.07	211	0.89

Panel C: RepRisk score: Social						
RepRisk Index	Sample	Average before investment	Average after investment	Difference	Observations	*p* value
RepRisk Social (1 year)	SDG	5.12	6.28	− 1.16	39	0.42
RepRisk Social (2 years)	SDG	4.62	6.02	− 1.41	27	0.33
RepRisk Social (3 years)	SDG	3.31	5.18	− 1.88	24	0.12
RepRisk Social (1 year)	Non-SDG	5.47	5.88	− 0.41	284	0.50
RepRisk Social (2 years)	Non-SDG	5.19	5.68	− 0.49	253	0.43
RepRisk Social (3 years)	Non-SDG	5.54	5.75	− 0.21	211	0.75

Panel D: RepRisk score: Governance						
RepRisk Index	Sample	Average before investment	Average after investment	Difference	Observations	*p* value
RepRisk Governance (1 year)	SDG	3.94	5.14	− 1.19	39	0.49
RepRisk Governance (2 years)	SDG	3.42	7.04	− 3.63*	27	0.09
RepRisk Governance (3 years)	SDG	2.36	7.36	− 5.01**	24	0.02
RepRisk Governance (1 year)	Non-SDG	6.24	8.06	− 1.83**	284	0.03
RepRisk Governance (2 years)	Non-SDG	5.79	9.27	− 3.48***	253	0.00
RepRisk Governance (3 years)	Non-SDG	5.25	10.39	− 5.14***	211	0.00

***, **, and * represent 1%, 5%, and 10% statistical significance levels respectively of the two-sided *t* test for the difference in means of the RepRisk Index before and after the investment

We then disentangle the total ESG score and unveil possible differences across sustainability pillars. Interestingly, the impact of SWF investment on the environmental performance of firms seems negligible. We report some evolution over time in the data, with an early improvement followed by a decline, but the comparison does not yield any systematic, statistically significant difference in firm behavior before and after the investment. A similar pattern surfaces by analyzing the social dimension.

Interestingly, most of the documented decline in the total ESG score can be attributed to the governance pillar. Indeed, as shown in Figure 9, the exposure to governance risk seems to follow almost a linear trend, worsening in absolute value and becoming more statistically significant as we extend the time horizon to 3 years, where it reaches the maximum. In this respect, the SDG and non-SDG subsamples do not behave differently, even if the deterioration is slightly more pronounced in the latter.

These descriptive statistics do not allow us to draw any causal inference about the negative impact of SWF investment on the corporate governance of target firms. Further analyses should consider the selection issues, endogeneity, and omitted variable bias possibly affecting these preliminary results. However, the observed decline in the G component of the ESG score is a well-established fact, providing some incomplete and preliminary support to H4b.

## POLICY RECOMMENDATIONS

SWFs as universal owners, namely institutional investors with large, globally diversified portfolios, are uniquely placed to foster and hasten the transition mentioned above, and genuinely deliver on the SDG. So far, however, as previously discussed in this article, they have adhered to a strict interpretation of their fiduciary duty, aligning their strategies to purely financial considerations, shunning any other goal that would make them look politically motivated. This approach was enshrined in the Santiago Principles, a voluntary high-level code of conduct that was drafted by the International Working Group of SWFs and endorsed by the International Monetary Fund in 2008. A review of these principles is probably overdue. The first policy recommendation that can be drawn from this paper is therefore the formulation of a “Santiago 2.0” version, led by the IFSWFs, where the pursuit of SDGs is explicitly stated as a legitimate goal and as an integral part of their fiduciary duty, catalyzing a broader adoption of sustainable investment practices among private and state-sponsored financial institutions alike.

A second policy recommendation is related to the finding in this paper, corroborating earlier research, that SWF investments in listed and unlisted firms correlate with deterioration in corporate governance in the investee company during SWF ownership. As noted in the "EMPIRICAL ANALYSIS" section, the data does not support causation and merits further investigation. A plausible hypothesis is that SWF’s may be more passive than other investors in the realm of governance, since they shy away from being seen to be politically motivated in their monitoring and oversight of investee strategy. This deterioration in governance then results in exacerbating agency costs and managerial complacency, which leads to a previously documented SWF discount in investment performance (Bortolotti et al., 2015). While more analysis is warranted, our policy recommendation is for SWFs to develop internal policies to strike a better balance between meaningfully contributing to investee corporate governance and remaining apolitical in society at large. Arguably, this balancing act involves trade-offs that currently seem to remain implicit and appear not to be well-thought through yet.

A third and final policy recommendation relates to the role of SWFs in making investments in climate change mitigation and adaptation at scale, or SDG 13. A total of 18 SWFs has so far signed up for the One Planet Sovereign Wealth Fund Coalition, along with several asset managers and private equity firms, under the aegis of the One Planet Summits initiated by President Macron of France. Its founding members were the SWFs of the UAE, Kuwait, Saudi Arabia, Qatar, Norway, and New Zealand. Outputs so far have included an ESG framework, annual ESG reporting, a One Planet Sovereign Wealth Fund Framework, and a Climate Disclosure Guidance for Private Markets. However, actions speak louder than words, and it is not clear if the SWF members of the Coalition have made any more investments in combating climate change, or at least reduced their investments in fossil fuel, than otherwise would have been the case. While momentum is building in climate investments by SWFs since 2018, especially in deal value, much more can be done, and much more is expected of the SWFs. Just as SWFs stepped up with large investments to combat the pandemic, SWFs may be called upon to invest in mitigating and adapting to an even greater global emergency, the climate emergency. A climate investment accord amongst SWFs could provide for meaningful and measurable investment commitments and outcomes.

These three policy recommendations, regarding fiduciary duty, investee corporate governance, and climate investments, would contribute to updating and modernizing the role of SWFs in the global economy, and make them fit-for-purpose for this decade.

## CONCLUSIONS

This article represents the first systematic attempt to quantitatively document the evolution of SWF sustainable investments over the last two decades. Our evidence – albeit purely descriptive – is broadly consistent with the consensus view of SWF as “sustainability laggards”: since the turn of the century they aligned only 16% of their deal count and 7% of their deal value with sustainable development. For those who espouse the normative belief that SWFs should engage deeply in making sustainable investments, these are, in an absolute sense, not overly impressive numbers.

But things are changing. Our data analysis reveals that over the last five years there has been a noticeable uptick in SDG investing by SWFs. From 2018 onwards, we see momentum building in climate and energy, especially in terms of deal value. We also see agriculture come to the fore in 2020, and to a lesser degree, investments in education, as the SWFs appear to slowly but surely extend their investment remits into other long-term investment themes.

While SWFs’ overall engagement in SDGs has been so far quite limited, the efforts displayed in the health industry are truly remarkable. With 249 deals worth almost USD 29 billion (altogether, without taking into account the winsorization), healthcare has been consistently over the period the SDG target sector of choice. Having backed the R&D efforts of many pharmaceutical firms that eventually developed COVID-19 vaccines, we can conclude that SWFs have played a key role in combating the pandemic.

Geographically, we see the deal value as well as the deal count of sustainable investments affected in MEASA, Europe, North America, and Asia–Pacific, in that order. While Europe and North America are attractive lower-risk OECD destinations, MEASA is a highly diverse region, exhibiting both high growth potential and socio-economic and environmental challenges and associated investment opportunities.

As it is often the case in SWF research, aggregate data mask important differences. Drilling further down to the level of individual SWFs, despite the documented reluctance at the industry level, we see a few emerging champions in sustainable investing. QIA, GIC, Temasek, ADIA, CIC, and Mubadala have each invested between USD 21.6 billion to USD 3.7 billion (not considering winsorization) aligned with SDGs. The healthcare sector is their predominant target of investments, invariably followed by a combination of energy, financial services, and infrastructure. All four sectors taken together typically make up around three quarters of their total deal count. Some smaller SWFs, such as Bahrain Mumtalakat and the New Zealand Superannuation Fund, report higher percentage of sustainable investments of their total investment activity, suggesting a consistent integration of sustainability in their investment operations.

An interesting question to ask is what will be the future SWFs’ stance in sustainable investment. As overarching themes such as climate change mitigation and adaptation and SDG-aligned investing become more and more prevalent in the institutional investment world, one might reasonably expect the SWFs, from their boards to their investment committees and officers, to align their investment approaches more and more with the SDGs.

Our analysis suggests that having solid ESG frameworks in place favors the execution of larger sustainable investment programs, indicating that SWFs ‘walk the way they talk’ when it comes to responsible investment. Interestingly, several key industry players have recently streamlined their ESG strategies. According to UNCTAD (2022), nearly three out of four reporting SWFs now have an impact investment strategy targeting thematic sectors, such as renewables, or use a specific ESG-related instrument, such as green bonds. We thus predict that this strategic revision will generate a more intense deal making by SWFs along the SDGs in the years to come.

More solid ESG policies in place and consequently more ESG-aligned investments are, however, only a necessary condition for driving real change and delivering on the sustainable development agenda. The academic literature to which this paper contributes to has shown that the track record of SWFs’ in pushing portfolio firms’ ESG performance is quite poor, so another key question to ask is whether “this time will be different”, namely whether the next wave of investment will generate meaningful social and environmental impact in the years to come.

Any prediction at this early stage could only be tentative, but we claim that achievements in societal performance of firms can be achieved if SWFs shift their position from passivity and institutional isolation from external pressures toward the acknowledgement of their global responsibilities in sustainable development.

The current multiple crises have painfully shown that neglecting environmental risks exposes both society and the economy to natural disasters, hurting the value of SWF assets and jeopardizing their obligations to their stakeholders. More generally, the realities of climate change, GFC, populist regimes and the pandemic, to name a few phenomena since the turn of the century, have brought the risks of a globally interconnected world into sharper focus, and have highlighted the need for a transition from the conventional model focused on short-term profits, shareholder value, and the dilapidation of natural capital, to a new paradigm in which environmental sustainability, social inclusion and shared prosperity should become central in corporate and financial decision-making.

## Notes

^1^This gap has been growing. The shortfall in financing for the SDGs has been exacerbated by the COVID-19 pandemic and its socio-economic impact in developing countries. The OECD’s 2020 Global Outlook on Financing for Sustainable Development projected that developing countries could face an additional shortfall of $1.7 trillion in financing in 2020. This would grow the existing annual financing gap of $2.5 trillion to an annual SDG financing gap of $4.2 trillion.

^2^The IRIS+ taxonomy has been developed by the Global Impact Investing Network (GIIN, 2021). See https://iris.thegiin.org/document/iris-thematic-taxonomy/.

^3^In a recent survey, asked whether the legislation governing their mandate limits their ability to further consider ESG factors, 30% of SWF responded positively (Invesco, 2021).

^4^Climate goals expose dilemma for wealth funds, *Financial Times*, October 27, 2021.

^5^The list of SWFs that are officially tracked stems from the SWF definition by Bocconi’s Sovereign Investment Lab (see Bortolotti et al., 2015).

^6^The small size of the SDG sub-samples could be responsible for the limited power of the test (see Cohen, Cohen, West, & Aiken, 2003).

^7^https://globalswf.com/.

^8^Global SWF (2022).

^9^Our approach allows to differentiate between ESG corporate disclosure and communications versus actual ESG practice. Indeed, the SUSTAINABILITY indicator derived from the Global SWFs GSR score reflects the first aspect as it captures whether the SWF has disclosed that an ESG risk management or ESG investment policy in place. The actual volume by number of deals and deal values of investments aligned with the SDG captures instead whether SWFs “walk the way they talk”, and by a revealed preference argument can be considered a proxy of one SWF’s commitment to sustainable investment. We are grateful to a referee for pointing out the need to clarify this distinction.

^10^We need to acknowledge that *every* healthcare deal fits within the IRIS+ definition of healthcare (either Access to Quality Healthcare or Nutrition) and that this inclusive bias therefore contributes significantly to the outsized position of the healthcare sector as a recipient of sustainable investments by the SWF community.

^11^https://www.statista.com/statistics/184968/us-health-expenditure-as-percent-of-gdp-since-1960/.

^12^An interesting research topic would be the analysis of the impact of large-scale SDG investments across asset classes on the overall SWFs’ performance, allowing to test whether sustainable investment is associated with concessionary or market returns. We thank an anonymous referee for this comment and leave this to further research due to current data limitations.

## Appendix 1: League Table by SWF SDG Deals

**Table:**

SWF	Sector								
	Agriculture	Biodiversity and Ecosystems	Climate	Education	Employment	Energy	Financial Services	Health	Infrastructure
Temasek Holdings Pte Ltd	17	20	5	6	1	10	21	113	13
GIC Pte Ltd	1	0	6	5	2	17	11	28	13
Mubadala Investment Company PJSC	0	0	2	6	0	14	0	21	3
Khazanah Nasional Bhd	0	0	0	2	2	4	2	19	3
Qatar Investment Authority	0	1	2	2	0	5	2	9	4
China Investment Corporation	0	0	0	0	1	5	3	9	1
Abu Dhabi Investment Authority	0	1	1	0	0	8	0	8	2
Ireland Strategic Investment Fund	1	1	0	0	0	2	2	11	0
New Zealand Superannuation Fund	0	0	0	0	0	4	2	3	0
Future Fund	0	0	0	1	1	0	0	6	2
Kuwait Investment Authority	0	0	0	1	0	1	1	6	0
Oman Investment Authority	0	0	0	0	0	2	2	3	1
Public Investment Fund	0	0	1	0	0	2	0	4	0
Russian Direct Investment Fund	0	1	0	0	0	1	1	5	0
Libyan Investment Authority	1	0	0	0	0	0	0	0	4
Bahrain Mumtalakat Holding Company	0	0	0	2	0	0	0	1	1
ADQ Holding Company PJSC	1	0	0	0	0	1	0	2	0
Government Pension Fund: Global	0	0	0	0	0	0	1	2	1
Korea Investment Corporation	0	0	1	0	0	1	0	0	1
State Oil Fund of the Republic of Azerbaijan	0	0	0	0	0	1	1	0	1
Investment Corporation of Dubai	2	0	0	0	0	0	0	0	0
Palestine Investment Fund	0	0	0	0	0	0	2	0	0
Brunei Investment Agency	0	0	0	0	0	0	0	1	0
Dubai International Financial Centre	0	0	0	0	0	0	0	0	0
Emirates Investment Authority	0	0	0	0	0	0	0	0	0
Fundo Soberano de Angola	0	0	0	0	0	0	0	0	0
Istithmar	0	0	0	0	0	0	0	0	0
Kazakhstan National Fund	0	0	0	0	0	0	0	0	0
National Social Security Fund	0	0	0	0	0	0	0	0	0
National Wealth Fund	0	0	0	0	0	0	0	0	0
RAK Investment Authority	0	0	0	0	0	0	0	0	0
State Capital Investment Corporation	0	0	0	0	0	0	0	0	0
Turkey Wealth Fund	0	0	0	0	0	0	0	0	0
Total	23	24	18	25	7	78	51	251	51

SWF	Sector								
	Land	Pollution	Real Estate	Waste	Water	SDG	Non SDG	% SDG	Total
Temasek Holdings Pte Ltd	0	1	3	4	2	216	602	26	818
GIC Pte Ltd	0	0	2	2	1	88	636	12	724
Mubadala Investment Company PJSC	0	0	0	3	4	51	284	15	335
Khazanah Nasional Bhd	0	0	0	0	1	33	76	30	109
Qatar Investment Authority	0	0	3	0	1	29	256	10	285
China Investment Corporation	0	0	0	0	2	21	252	8	273
Abu Dhabi Investment Authority	0	0	0	0	0	20	157	11	177
Ireland Strategic Investment Fund	0	0	1	1	0	19	35	35	54
New Zealand Superannuation Fund	1	1	0	1	0	12	34	26	46
Future Fund	0	0	0	0	0	10	47	18	57
Kuwait Investment Authority	0	0	0	0	1	10	110	8	120
Oman Investment Authority	0	0	1	0	0	9	73	11	82
Public Investment Fund	0	0	0	1	0	8	32	20	40
Russian Direct Investment Fund	0	0	0	0	0	8	42	16	50
Libyan Investment Authority	0	0	1	0	0	6	47	11	53
Bahrain Mumtalakat Holding Company	0	0	0	0	1	5	11	31	16
ADQ Holding Company PJSC	0	0	0	0	0	4	12	25	16
Government Pension Fund: Global	0	0	0	0	0	4	61	6	65
Korea Investment Corporation	0	0	0	0	0	3	29	9	32
State Oil Fund of the Republic of Azerbaijan	0	0	0	0	0	3	27	10	30
Investment Corporation of Dubai	0	0	0	0	0	2	20	9	22
Palestine Investment Fund	0	0	0	0	0	2	1	67	3
Brunei Investment Agency	0	0	0	0	0	1	23	4	24
Dubai International Financial Centre	0	0	0	0	0	0	14	0	14
Emirates Investment Authority	0	0	0	0	0	0	4	0	4
Fundo Soberano de Angola	0	0	0	0	0	0	4	0	4
Istithmar	0	0	0	0	0	0	2	0	2
Kazakhstan National Fund	0	0	0	0	0	0	3	0	3
National Social Security Fund	0	0	0	0	0	0	37	0	37
National Wealth Fund	0	0	0	0	0	0	2	0	2
RAK Investment Authority	0	0	0	0	0	0	4	0	4
State Capital Investment Corporation	0	0	0	0	0	0	2	0	2
Turkey Wealth Fund	0	0	0	0	0	0	11	0	11
Total	1	2	11	12	13	564	2,950	16	3,514

Data presented in the table is not winsorized

## Appendix 2: League Table by SWF SDG Deals (USD millions)

**Table:**

SWF	Sector								
	Agriculture	Biodiversity and Ecosystems	Climate	Education	Employment	Energy	Financial Services	Health	Infrastructure
Qatar Investment Authority	0	16	229	217	0	1,919	362	3,380	15,150
GIC Pte Ltd	22	0	110	1,064	375	3,585	659	7,679	877
Temasek Holdings Pte Ltd	855	529	150	490	10	230	312	9,596	1,080
Abu Dhabi Investment Authority	0	1,250	625	0	0	1,171	0	3,541	5
Mubadala Investment Company PJSC	0	0	129	6	0	3,856	0	1,312	566
China Investment Corporation	0	0	0	0	0	2,405	900	316	100
Khazanah Nasional Bhd	0	0	0	11	127	35	53	1,591	419
New Zealand Superannuation Fund	0	0	0	0	0	205	59	319	0
Public Investment Fund	0	0	1,000	0	0	0	0	139	0
Future Fund	0	0	0	34	5	0	0	244	348
Kuwait Investment Authority	0	0	0	0	0	4	3	486	0
Ireland Strategic Investment Fund	0	59	0	0	0	17	20	275	0
Oman Investment Authority	0	0	0	0	0	118	24	150	113
State Oil Fund of the Republic of Azerbaijan	0	0	0	0	0	50	109	0	86
Libyan Investment Authority	30	0	0	0	0	0	0	0	171
Bahrain Mumtalakat Holding Company	0	0	0	0	0	0	0	45	170
Investment Corporation of Dubai	103	0	0	0	0	0	0	0	0
Korea Investment Corporation	0	0	10	0	0	1	0	0	50
Palestine Investment Fund	0	0	0	0	0	0	60	0	0
Government Pension Fund - Global	0	0	0	0	0	0	0	13	4
Russian Direct Investment Fund	0	9	0	0	0	0	0	5	0
ADQ Holding Company PJSC	0	0	0	0	0	0	0	0	0
Brunei Investment Agency	0	0	0	0	0	0	0	0	0
Dubai International Financial Centre	0	0	0	0	0	0	0	0	0
Emirates Investment Authority	0	0	0	0	0	0	0	0	0
Fundo Soberano de Angola	0	0	0	0	0	0	0	0	0
Istithmar	0	0	0	0	0	0	0	0	0
Kazakhstan National Fund	0	0	0	0	0	0	0	0	0
National Social Security Fund	0	0	0	0	0	0	0	0	0
National Wealth Fund	0	0	0	0	0	0	0	0	0
RAK Investment Authority	0	0	0	0	0	0	0	0	0
State Capital Investment Corporation	0	0	0	0	0	0	0	0	0
Turkey Wealth Fund	0	0	0	0	0	0	0	0	0
Total	1,011	1,864	2,252	1,823	517	13,594	2,561	29,091	19,140

SWF	Sector								
	Land	Pollution	Real Estate	Waste	Water	SDG	Non SDG	% SDG	Total
Qatar Investment Authority	0	0	260	0	0	21,533	128,930	14	150,463
GIC Pte Ltd	0	0	36	36	135	14,578	157,028	8	171,606
Temasek Holdings Pte Ltd	0	0	3	28	120	13,404	95,883	12	109,287
Abu Dhabi Investment Authority	0	0	0	0	0	6,592	47,611	12	54,203
Mubadala Investment Company PJSC	0	0	0	29	583	5,974	123,580	5	129,554
China Investment Corporation	0	0	0	0	45	3,765	172,564	2	176,329
Khazanah Nasional Bhd	0	0	0	0	0	2,236	13,595	14	15,831
New Zealand Superannuation Fund	626	60	0	65	0	1,334	1,657	45	2,992
Public Investment Fund	0	0	0	0	0	1,139	58,473	2	59,612
Future Fund	0	0	0	0	0	632	7,211	8	7,843
Kuwait Investment Authority	0	0	0	0	0	492	30,967	2	31,459
Ireland Strategic Investment Fund	0	0	31	26	0	428	14,124	3	14,552
Oman Investment Authority	0	0	0	0	0	405	4,609	8	5,014
State Oil Fund of the Republic of Azerbaijan	0	0	0	0	0	244	2,962	8	3,206
Libyan Investment Authority	0	0	20	0	0	222	5,249	4	5,470
Bahrain Mumtalakat Holding Company	0	0	0	0	0	215	200	52	414
Investment Corporation of Dubai	0	0	0	0	0	103	7,027	1	7,130
Korea Investment Corporation	0	0	0	0	0	61	3,995	2	4,056
Palestine Investment Fund	0	0	0	0	0	60	0	100	60
Government Pension Fund - Global	0	0	0	0	0	18	18,264	0	18,281
Russian Direct Investment Fund	0	0	0	0	0	13	2,029	1	2,042
ADQ Holding Company PJSC	0	0	0	0	0	0	2,184	0	2,184
Brunei Investment Agency	0	0	0	0	0	0	1,117	0	1,117
Dubai International Financial Centre	0	0	0	0	0	0	2,867	0	2,867
Emirates Investment Authority	0	0	0	0	0	0	6,824	0	6,824
Fundo Soberano de Angola	0	0	0	0	0	0	180	0	180
Istithmar	0	0	0	0	0	0	250	0	250
Kazakhstan National Fund	0	0	0	0	0	0	406	0	406
National Social Security Fund	0	0	0	0	0	0	12,571	0	12,571
National Wealth Fund	0	0	0	0	0	0	2,472	0	2,472
RAK Investment Authority	0	0	0	0	0	0	12	0	12
State Capital Investment Corporation	0	0	0	0	0	0	95	0	95
Turkey Wealth Fund	0	0	0	0	0	0	11,325	0	11,325
Total	626	60	351	183	883	73,449	936,260	7	1,009,709

Data presented in the table is not winsorized
